# The role of RNA methyltransferase METTL3 in gynecologic cancers: Results and mechanisms

**DOI:** 10.3389/fphar.2023.1156629

**Published:** 2023-03-16

**Authors:** Yuxiang Zhang, Na Zhang

**Affiliations:** ^1^ Department of Radiation Oncology, Cancer Hospital of China Medical University, Liaoning Cancer Hospital and Institute, Shenyang, China; ^2^ Department of Cancer Hospital, China Medical University, Shenyang, China

**Keywords:** N6‐methyladenosine, m6A methyltransferase, gynecologic malignancies, METTL3, cancer therapy

## Abstract

N6-methyladenosine (m6A) methylation is the most prevalent mRNA modification in eukaryotes, and it is defined as the methylation of nitrogen atoms on the six adenine (A) bases of RNA in the presence of methyltransferases. Methyltransferase-like 3 (Mettl3), one of the components of m6A methyltransferase, plays a decisive catalytic role in m6A methylation. Recent studies have confirmed that m6A is associated with a wide spectrum of biological processes and it significantly affects disease progression and prognosis of patients with gynecologic tumors, in which the role of Mettl3 cannot be ignored. Mettl3 is involved in numerous pathophysiological functions, such as embryonic development, fat accumulation, and tumor progression. Moreover, Mettl3 may serve as a potential target for treating gynecologic malignancies, thus, it may benefit the patients and prolong survival. However, there is a need to further study the role and mechanism of Mettl3 in gynecologic malignancies. This paper reviews the recent progression on Mettl3 in gynecologic malignancies, hoping to provide a reference for further research.

## Introduction

Gynecologic malignancies are major diseases that pose a threat to the health of women worldwide; and among them, cervical cancer (CC), ovarian cancer (OC), and endometrial cancer (EC) are the most common malignancies. In recent years, patients diagnosed with these diseases are more likely to be younger (Bray et al., 2018). Surgical intervention is the primary treatment for gynecological malignancies and is often complemented with radiotherapy, chemotherapy, endocrine therapy, and immunotherapy (Barillot and Haie-Meder, 2009; Wang et al., 2020a; Chen et al., 2022). Despite these treatment modalities, the overall prognosis for patients with gynecological malignancies remains unsatisfactory, indicating the need for further improvements in therapeutic strategies. The current treatment regimen faces many challenges due to the high malignancy, high mortality rate, and high incidence of recurrence and metastasis in gynecologic cancers (Jemal et al., 2011; Makker et al., 2020; Lim et al., 2022). Therefore, it is imperative to explore the detailed molecular mechanisms of gynecologic tumors and to seek innovative therapeutic strategies. Gynecological malignancies result from multiple pathogenic factors, among which abnormal genetic changes in proto-oncogenes and tumor suppressor genes, such as gene expansion/deletion/mutation or chromosomal translocation, are essential factors in the initiation and progression of tumors (Dueñas-González et al., 2005; See et al., 2019; Zhou et al., 2016). Most previous reports have focused on the DNA expression level; but with the progress of research, it has become clear that regulation at the transcriptional level is equally essential. Mounting evidence has revealed that epigenetic modifications regulate gene expression without altering the DNA sequence and always modulate tumorigenesis and progression (Nebbioso et al., 2018; Zhu et al., 2021). Among the various epigenetic modifications, m6A methylation modification is the most frequent and significant modification in messenger RNA (Liang et al., 2020a).

**FIGURE 1:**
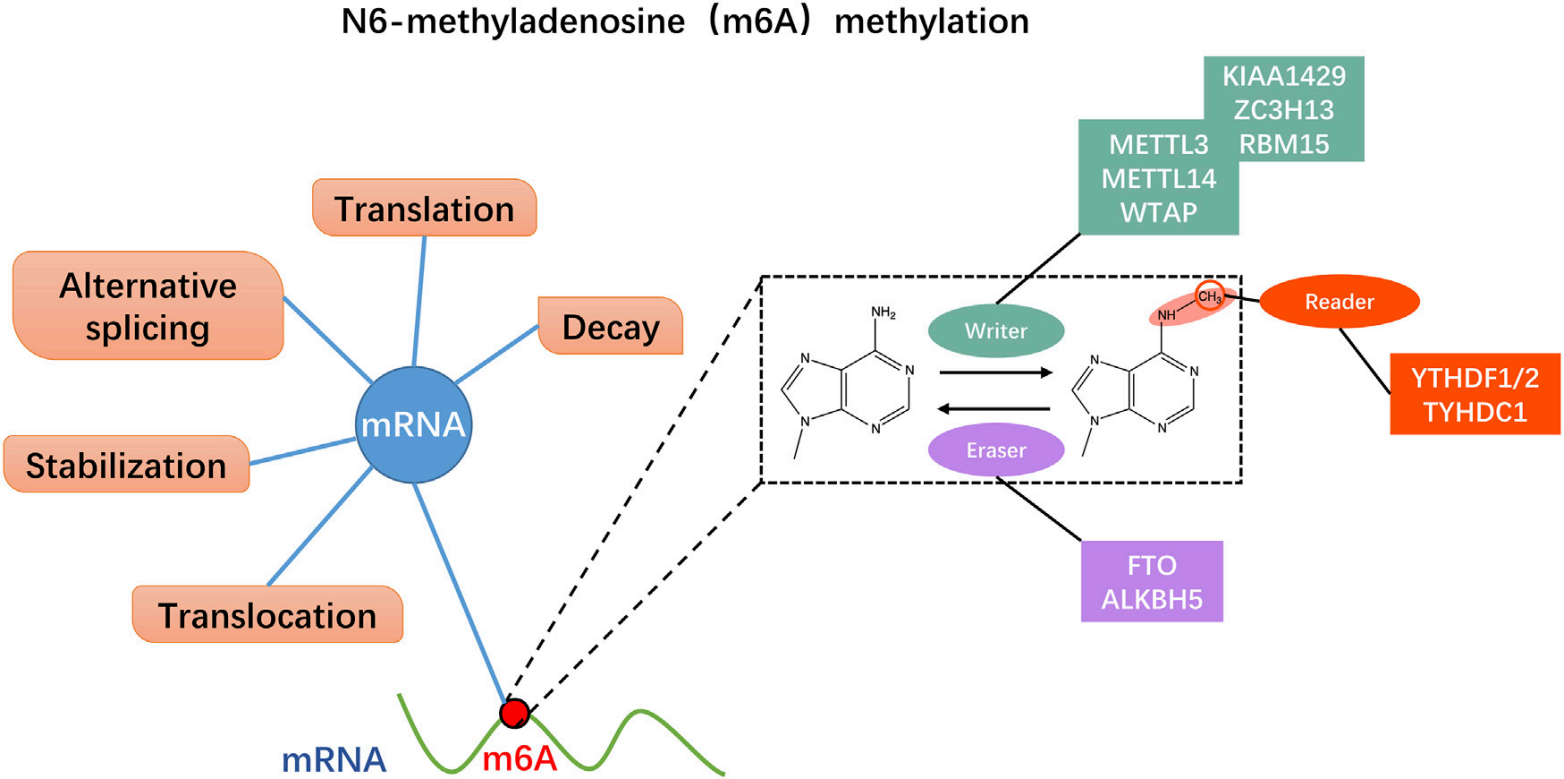
N6-methyladenosine (m6A) methylation dynamically regulates mRNA through multiple pathways. M6A regulatory proteins include m6A methyltransferases (m6A writers), m6A demethylases (m6A erasers), and m6A recognition proteins (m6A readers). The writers act as methyltransferase including Mettl3/14 and WTAP. The erasers mediate demethylation modifications, like FTP and ALKBH5. M6A readers recognize information about mRNA methylation and are involved in downstream mRNA translation, degradation, and other behaviors, such as YTHDF1/2 and TYHDC1.

N6-methyladenosine (m6A) modification in RNA is defined as the methylation of nitrogen atoms on the six adenine (A) bases of RNA in the presence of methyltransferases (Huang et al., 2021a). Although m6A methylation was identified in poly(A) RNA from hepatocellular carcinoma cells in 1974 (Desrosiers et al., 1974), researchers used methylated RNA immunoprecipitation sequencing (MeRIP-Seq) in 2012 to revive interest in m6A methylation (Dominissini et al., 2012; Meyer et al., 2012). With increasing research and advent of high-throughput sequencing technology, understanding of m6A methylation has gradually improved. A growing number of studies have confirmed that m6A methylation is closely related to various biological behaviors of malignant tumors and affects patient prognosis (Huang et al., 2022). M6A methylation is a multi-stage process resulting from the interaction of three enzymes: methyltransferases (m6A writers), demethylases (m6A erasers), and binding proteins (m6A readers) (Figure 1) (Helm and Motorin, 2017; Pinello et al., 2018). Among them, m6A methyltransferases mainly include methyltransferase-like 3 (Mettl3), methyltransferase-like 14 (Mettl14), and Wilms tumor one associated protein (WTAP) (Lan et al., 2019); M6A demethylase specifically includes alkB homolog 5 (ALKBH5) and fat mass and obesity-associated protein (FTO) (Chen et al., 2019); YT521‐B homology (YTH) domain family proteins comprising YTHDFs and YTHDCs mainly constitute m6A binding proteins. The YTHDF subtype family consists of YTHDF1, YTHDF2, and YTHDF3, while the YTHDC subtype family mainly comprises YTHDC1 and YTHDC2 (Zhao et al., 2020a). Heterogeneous nuclear ribonucleoproteins (HNRNPs) and insulin-like growth factor 2 mRNA-binding proteins (IGF2BPs) are also crucial compositions of m6A readers. The role of m6A methylation serving as a “double-edged sword” in malignancy can be complex and multifaceted. On the one hand, m6A methylation has been shown to promote tumorigenesis and progression by regulating various cellular processes such as RNA stability, splicing, translation, and protein-protein interactions (Fang et al., 2022). On the other hand, m6A methylation can also exert cancer-suppressive effects by controlling the expression of tumor suppressor genes and inhibiting oncogenic signaling pathways (Deng et al., 2022). The regulatory role of m6A methylation in malignancy likely varies depending on the specific type of cancers, the stage of cancer development, and other factors (Liu et al., 2018a). As an indispensable regulator of m6A methylation, Mettl3 is a critical protein in the m6A methyltransferase complex, which catalyzes methylation of the nitrogen atom at position 6 of adenine (A) in RNA. Mettl3, Mettl14, and accessory proteins, including WTAP and ZC3H13, constitute the m6A writer proteins, and Mettl3 is the only catalytic subunit that uses S-adenosylmethionine (SAM) as a methyl donor (Wang et al., 2016a; Wang et al., 2016b). Mettl3 is highly conserved in eukaryotes and is a vital component of the m6A methyltransferase complex, which plays a crucial catalytic role in the entire process of m6A methylation (Wang et al., 2016b). Also, Mettl3 is engaged in almost all RNA stages involving m6A methylation, including pre-mRNA splicing, mRNA decay, miRNA processing, translation regulation, and nuclear export (Wang et al., 2020b).

Therefore, Mettl3 may be a new target for treating gynecological malignancies. Conducting a comprehensive investigation into the specific mechanism of Mettl3 in gynecologic malignancies could provide substantial benefits to a diverse patient population by facilitating the development of effective diagnostic and therapeutic strategies. This article reviews the latest research progress of Mettl3 in gynecologic malignancies, as well as the detailed mechanisms behind, to offer insights into innovation in the diagnosis and treatment of gynecologic malignancies.

## Molecular structure, the primary function of Mettl3

**FIGURE 2:**
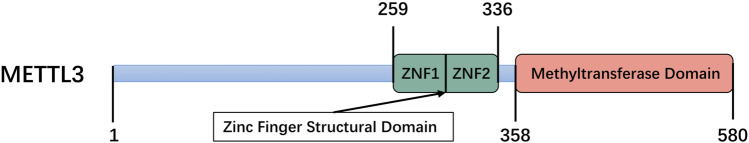
Schematic domain structure of Mettl3, including zinc finger domain (ZFD) and methyltransferase domain (MTD).

Mettl3 comprises a zinc finger structural domain (ZFD) and a methyltransferase domain (MTD) construct containing 358–580 residues, both of which are essential for enzymatic activity (Figure 2) (Wang et al., 2016a). The MTD of the Mettl3-Mettl14 heterodimer has been identified by investigators using X-ray crystallographic methods. Mettl3 represents the only catalytic subunit that binds to the methyl donor SAM and facilitates the transfer of methyl groups (Wang et al., 2016a; Zeng et al., 2020). However, MTD alone cannot ensure an enzymatically active Mettl3-mettl14 complex and participation of ZFD is required to obtain a fully functional complex (Śledź and Jinek, 2016). Huang et al. used magnetic resonance to reveal that the ZFD solution structure specifically binds to RNA containing the 5′-GGACU-3′ shared sequence but not to RNA without this sequence, and that it contains two CCCH-type zinc fingers linked in tandem by an inverse parallel ß-sheet linkage while having a synergistic catalytic effect with MTD3-MTD14 (Huang et al., 2019). The impact of Mettl3 on tumor progression is mediated through numerous mechanisms. In breast cancer, the pro-oncogenic function of Mettl3 involves the inhibition of let-7g tumor suppressor translation, which ultimately promotes cancer cell proliferation (Cai et al., 2018). Wang et al. confirmed that Mettl3 regulates VASH1 to promote brain metastasis in lung cancer by inducing the maturation of miR-25-3p (Wang et al., 2019). Mettl3 promotes hepatocellular carcinoma cell proliferation and metastasis by reducing the mRNA stability of SOCS2 through a YTHDF2-dependent pathway (Chen et al., 2018; Wang et al., 2020c). In short, Mettl3 exerts a significant and multifaceted biological impact on diverse cancer types and all stages of tumorigenesis (Yi et al., 2020). While the role of Mettl3 in tumorigenesis has been extensively studied in recent years, there is still much to learn about its mechanisms of action and potential as a therapeutic target. As research continues, it is likely that more insights will be gained into the complex and integrative role that Mettl3 plays in cancer.

## Advances of Mettl3 in various gynecological malignancies

**FIGURE 3:**
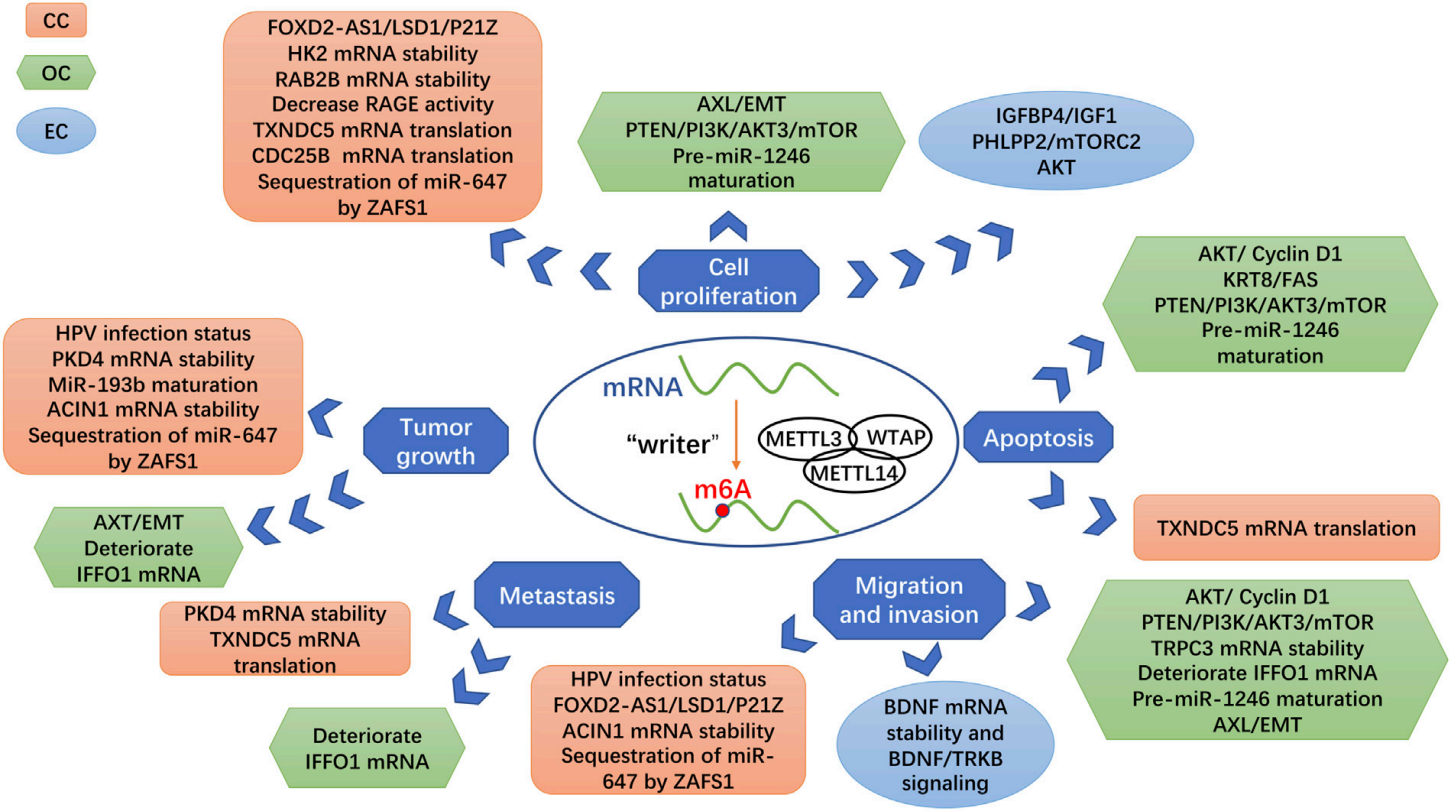
Roles of Mettl3 as a methyltransferase in gynecologic cancers. Different targets of Mettl3 are linked with various signaling pathways and biological processes, which generate the different influences of Mettl3 in gynecologic cancers.

Previous investigations have shown that Mettl3 plays a critical role in promoting cancer development in diverse tissue types of cancer, such as affecting the development of gastrointestinal malignancies by affecting tumor proliferation, angiogenesis, apoptosis, and metastasis (Figure 3) (Wang et al., 2020c). Analogously, Mettl3 is closely associated with multiple adverse biological behaviors of gynecologic malignancies, including tumor proliferation, apoptosis, metastasis, angiogenesis, and immune microenvironment (Huang et al., 2022). We reviewed the latest research on the mechanism of Mettl3 in the occurrence and development of gynecological malignancies. Therefore, exploring the specific mechanism of m6A methylation in malignant tumors is very important for attacking cancer.

## The role of Mettl3 in cervical cancer

CC has the fourth highest incidence and mortality rate among women, constituting a significant public health problem that endangers women’s health globally (Bray et al., 2018), (Cohen et al., 2019). According to global cancer statistics for 2018, an estimated 570,000 new cases of CC were reported, with approximately 311,000 related deaths (Bray et al., 2018). Hence, further research into the specific molecular mechanisms of CC is fundamental and it may provide inspiration for new therapeutic targets. It has been well established that infection with human papillomavirus (HPV), especially high-risk types, such as 16 and 18, is a high-risk factor for developing CC (Burd, 2003; Ma and Yang, 2021). Despite the increasing prevalence of HPV vaccination, HPV infection-associated CC remains a clinical challenge. Human papillomaviruses can produce single-stranded circular RNAs (circRNAs) containing the E7 oncogene (circE7). CircE7 is modified by m6A methylation and translated to produce the E7 oncoprotein, which promotes the growth of CC cells (Zhao et al., 2019). The regulatory protein of m6A methylation is closely linked to the HPV infection status of patients with CC, as it affects the extent of m6A methylation modification (Condic et al., 2022). Mettl3 is significantly overregulated in HPV-positive CC tissues and correlates with immune checkpoints and infiltration (Table 1) (Yu et al., 2022). The relationship between m6A methylation and HPV infection-associated CC needs further investigation. Addressing the etiological factors of CC can provide a treatment approach that targets the underlying cause, leading to significant clinical benefits for prevention and treatment.

**TABLE 1:** The role of Mettl3 in cervical cancer.

Cancer species	Expression	Biological function	Mechanism	Reference
cervical cancer	Up	Promote cell migration, chemotaxis, and tumor growth, influence cytokines	Associated with HPV infection status, immune checkpoint molecules, and immune infiltration	Yu et al. (2022)
	Up	Promote tumor growth, metastasis and chemoresistance	Regulate glycolysis in cervical cancer cells by enhancing the mRNA stability of PDK4	Li et al. (2020)
	Up	Promote cell proliferation and migration	Downregulate p21 through FOXD2-AS1/LSD1/P21*Z* axis	Ji et al. (2021)
	Up	Promote cell proliferation and warburg effect	Recruit YTHDF1 to enhance the stability of HK2 mRNA	Wang et al. (2020d)
	Down	Increase cisplatin chemosensitivity, inhibit the viability and proliferation	Decrease the activity of RAGE	Li et al. (2021a)
	Up	Promote cell proliferation and metastasis and inhibit apoptosis and autophagy	Promote the translation of TXNDC5 mRNA	Du et al. (2022)
	Up	Associated with poor prognosis, and promote cell proliferation	Increase mRNA stability of oncogene RAB2B *via* GF2BP3-dependent pathway	Hu et al. (2020)
	Up	Independent indicators for poor prognosis	The expression of Mettl3 was positively related with iASPP	Wu et al. (2020)
	Up	Promote tumor development, and associated with poor outcomes	The level of Mettl3 is positively linked to the number of CD33^+^ MDSC	Ni et al. (2020)
	Up	Confer cancer cell tolerance to hypoxic stress, promoting cell survival and adaptation	Enhance cytoprotective autophagy and tolerance to hypoxic environments by increasing the mRNA stability of DARS and acting on the DARS-AS1/ATG5/ATG3 axis	Shen et al. (2022a)
	Up	Facilitate cell cycle progression, promote cell proliferation *in vitro* and enhance tumorigenicity *in vivo*	Accelerated the translation of CDC25B mRNA through YTHDF1-dependent m6A modification	Li et al. (2022)
	Up	Promote tumor growth	Hinder miR-193b maturation, which inhibits tumorigenesis of cervical cancer cells through CCND1 targeting	Huang et al. (2021b)
	Up	Promote tumor growth, migration and aggressiveness	Mettl3 interacts with IGF2BP3 to promote the mRNA stability of ACIN1	Su et al. (2022)
	Up	Associated with poor prognosis	Negatively correlated with the expression of PD-L1	Ji et al. (2022)
	Up	Promote tumor growth *in vivo* and cell proliferation, migration, and invasion *in vitro*	Promote the sequestration of miR-647 by ZAFS1 through the regulation of RNA-RNA interactions	Yang et al. (2020)

In addition to its association with the HPV infection status of CC patients, Mettl3 plays a profound role in various biological behaviors of CC cells, exerting significant effects. *In vitro*, transcription is activated due to TATA-binding protein (TBP) binding to the Mettl3 promoter to the point at which Mettl3 expression levels are significantly increased (Li et al., 2020). Overexpression of Mettl3 promotes cancer progression through various pathways, such as affecting glucose metabolism, cell cycle PD-L1, immune infiltration, and enhancing the stability of oncogenic mRNA to promote the proliferation and migration of CC cells (Ji et al., 2022). Expression of Mettl3 is an independent risk factor for the 5-year recurrence-free survival (RFS), distant metastasis-free survival (DMFS), progression-free survival (PFS), and overall survival (OS) in patients with CC (Wu et al., 2020). Mechanically, the level of Mettl3 in tumor cells and tumor-infiltrating immune cells potentiate the density of myeloid-derived suppressor cells (MDSCs) and attenuate the proliferation of T lymphocytes in the tumor microenvironment, as well as hinder their function (Ni et al., 2020). Fei Ji et al. found that Mettl3 was positively correlated with the expression and enhanced the stability of the oncogene FOXD2-AS1, which promoted the proliferation and migration ability of CC cells while inhibiting apoptosis. In addition, the upregulated expression of the FOXD2-AS gene induced by Mettl3 recruits lysine-specific demethylase 1 (LSD1) to the P21 promoter region, which constitutes the FOXD2-AS1/LSD1/P21*Z* axis and reduces the expression of P21, an essential member of the cell cycle protein-dependent kinase inhibitor family. P21 is closely related to tumor suppression. Its overexpression inhibits the proliferation and migration ability of CC cells and enhances the apoptosis rate (Ji et al., 2021). In addition, Mettl3 synergizes with IGF2BP3 to extend the half-life of ACIN1 and RAB2B mRNA to contribute to the growth of CC and poor prognosis (Hu et al., 2020; Su et al., 2022). Recent research has revealed that Mettl3 is highly expressed in CC cells in the M stage, increases m6A methylation of CDC25B mRNA, and enhances its translation activity by recruiting YTHDF-1. Ultimately, the high expression level of Mettl3 further facilitates the cell cycle and proliferation (Li et al., 2022). Studies have provided additional evidence supporting the notion that Mettl3-mediated m6A methylation modifications result in the sequestration of miR-647 by ZAFS1 through the regulation of RNA-RNA interactions. This sequestration has been found to promote tumor growth *in vivo* and CC cell proliferation, migration, and invasion *in vitro*. Moreover, elevated levels of ZAFS1 have been clinically associated with lymph nodes and distant metastasis in CC patients and have been shown to be a predictor of poor prognosis (Yang et al., 2020). The profound involvement of Mettl3 in the diverse malignant biological behaviours plays a broad and remarkable role in the progression of CC cells.

Tumor cells exhibit aberrant metabolism, a distinct hallmark feature that facilitates their nutrient requirements for growth and proliferation, distinguishing them from normal cells (Pavlova and Thompson, 2016). The process of m6A methylation has been closely linked to the metabolic reprogramming of tumor cells (An and Duan, 2022). The Warburg effect, also known as aerobic glycolysis, is a typical abnormal metabolic behavior of tumors, which is an epiphenomenon of the transformation process crucial for cancer growth (Heiden et al., 2009; Spencer and Stanton, 2019). Although glycolysis is inefficient energy harvesting, it provides tumor cells with unique growth advantages, such as promoting tumor metastasis. Lactate produced in this process also helps tumor cells escape immunity and induces the secretion of vascular endothelial growth factors to provide nutrients to the tumor (Liberti and Locasale, 2016). In CC, suppressed Mettl3 causes a decrease in the oxygen consumption rate (OCR) and an increase in the extracellular acidification rate (EACR) and ATP generation efficiency (Li et al., 2020). Mettl3 targets the m6A site of the 3′-untranslated region (3′-UTR) of hexokinase 2 (hk2) mRNA and recruits YTHDF1 to enhance the stability of HK2 mRNA, further promoting the Warburg effect (Wang et al., 2020d). Therefore, Mettl3 may serve as a potential target to inhibit aerobic glycolysis by acting on the key enzyme of glycolysis-hexokinase, further delaying CC progression. In addition, studies have demonstrated that Mettl3-induced m6A modification leads to increased TXNDC5 expression in CC cells. Mechanistically, Mettl3 inhibits the ER stress that triggers apoptosis and autophagy by targeting TXNDC5 (Du et al., 2022). Consequently, Mettl3 regulates PDK4 mRNA stability and translation prolongation in CC cells through 5′UTR, ultimately affecting glycolysis. The authors also found that IGF2BP3 and ALKBH5 are involved in regulating m6A expression in CC cells and are associated with CC cell growth and chemosensitivity (Li et al., 2020). Under hypoxic conditions, Mettl3/Mettl14 is also bound to DARS mRNA, enhancing its m6A modification and reinforcing its stability. This resulted in the upregulation of DARS expression and mediated hypoxia-induced autophagy in CC cells *via* the DARS-AS1/ATG5/ATG3 axis, strengthening the degree of cellular tolerance to the hypoxic environment and promoting cell survival (Shen et al., 2022a). Tumor metabolic reprogramming plays a pivotal role in the infinite proliferative growth of tumors, an extremely complex process that encompasses more than just the Warburg effect (DeBerardinis et al., 2008). In addition to the Warburg effect, the connections between the glutamine metabolic pathway, serine synthesis, oxidative phosphorylation, and pentose phosphate pathway with m6A methylation and Mettl3 need to be urgently investigated.

The m6A methylation regulation has an extensive and multifaceted impact on tumorigenesis. While Mettl3 plays a promoting role in CC, it also inhibits the progression of CC cells through multiple mechanisms. Ruyi Li et al. proved that Mettl3 exhibited the opposite effect in CC (Li et al., 2021a). This study showed that Mettl3 is highly upregulated in paracancerous tissues of CC and inhibited tumor cell proliferation and viability. In the Mettl3-overexpressed SiHa cells line, researchers found that deletion of Mettl3 can reduce the apoptosis rate of CC cells and lead to high expression of drug resistance-related proteins (MRP1 and LRP1). Meanwhile, Mettl3 inhibited the expression of the known oncogene RAGE, which has been confirmed to promote cell proliferation and inhibit apoptosis in CC cells, thereby increasing the sensitivity of CC cells to cisplatin (Li et al., 2021a). Also, Mettl3 regulates epigenetic silencing of miR-193b, which is associated with CC tumorigenesis. Further studies have proved that the tumor suppressor effect of miR-193b occurs through diminishing CCND1, a cell cycle regulator associated with cyclin-dependent kinase 4 or 6 (CDK4/6). The role of CDK4/6 in promoting cell cycle progression from the G0–G1 phase to the S and G2-M phases has been confirmed (Huang et al., 2021b).

Overall, high expression of Mettl3 in CC is associated with poor prognosis, but some studies have suggested that Mettl3 is beneficial in inhibiting tumor progression. The dual regulatory function of Mettl3 in CC poses an intriguing scientific inquiry, whether it arises from the heterogeneity of tumor cells, the distinctive biological properties of Mettl3, or other underlying factors. Although the function of Mettl3 in CC has been partially explored, the role of Mettl3 in CC is still complex; and it remains to be further explored by investigators in terms of deeper mechanisms.

## The role of Mettl3 in ovarian cancer

OC, a silent killer, has the third highest incidence rate among all gynecologic malignancies; however, it has the highest mortality rate due to its extremely insidious early symptoms and often causes distant metastases when detected (Torre et al., 2018; Nameki et al., 2021). A woman has close to a one in 70 chance of developing OC during her lifespan, with an estimated 308,069 OC cases and 193,811 cases of OC mortality worldwide in 2020 (Smith, 2017). Therefore, early detection and effective treatment options for OC are imperative research areas that can significantly improve patient outcomes. Extensive investigations have demonstrated the crucial involvement of Mettl3 in OC pathogenesis, as it significantly promotes the growth and invasion of OC cells through various mechanisms, including modulation of the PI3K/AKT signaling pathway (Chang et al., 2021). Thus, thorough investigations of the molecular mechanisms underlying m6A methylation in ovarian tissues may uncover novel avenues for the diagnosis and treatment of OC.

The present studies found that Mettl3 functions as an oncogene in OC and is closely associated with various malignant behaviors of OC cells. Research indicates that the expression level of Mettl3 was markedly elevated in cancerous ovarian tissues compared to adjacent normal tissues, as demonstrated in a sample cohort of 75 OC patients. Highly expressed Mettl3 promotes miR-126-5p maturation by increasing the degree of m6A modification of pri-miR-126-5pand facilitates tumorigenesis and migration through the PTEN/PI3K/AKT3/mTOR axis (Table 2) (Bi et al., 2021a). In addition, Ma et al. collected tumors and adjacent tissues of 33 endometrioid epithelial ovarian cancer (EEOC) patients. Similarly, Mettl3 was found to be highly expressed in cancerous tissues, and overexpression of Mettl3 was an independent risk factor for higher tumor grade as well as for lower overall survival. The authors reconfirmed *in vitro* that Mettl3 could promote the proliferation, invasion, and migration of EEOC cells and it was not affected by the expression of Mettl14 and WTAP. Moreover, low expression of Mettl3 decreased the degree of m6A methylation of OC-related genes EIF3C, AXL, and CSF-1 (Ma et al., 2020). Importantly, Mettl3 also promotes the conversion of pre-miR-1246 to mature miR-1246 mRNA. In turn, miR-1246 targets CCNG2 and inhibits its expression, ultimately leading to OC cell proliferation, migration, and invasion; and inhibition of apoptosis (Bi et al., 2021b). It has been demonstrated that PLAA induces Mettl3 degradation by elevating the level of Mettl3 ubiquitination. Low levels of Mettl3 decreased the m6A methylation level of TRPC3 mRNA and inhibited TRPC3 expression, which resulted in lower intracellular calcium levels and suppressed OC cell metastasis (Shen et al., 2022b). Meanwhile, the Mettl3/YTHDF2 axis represses tumor suppressor gene IFFO1 expression and promotes mRNA degradation *via* an m6A methylation-dependent pathway, ultimately affecting the growth and development, metastasis and invasion, and cisplatin sensitivity of OC cells (Zhang et al., 2023).

**TABLE 2:** The role of Mettl3 in ovarian cancer.

Cancer species	Expression	Biological function	Mechanism	Reference
ovarian cancer	Up	Promote cell proliferation, migration, and invasion, and inhibit apoptosis	Promote miR-126-5p maturation by increasing the degree of m6A modification of pri-miR-126-5pmRNA and facilitate tumorigenesis and migration through the PTEN/PI3K/AKT3/mTOR axis	Bi et al. (2021a)
	Up	Promote proliferation, migration, invasion, and inhibition of apoptosis	Promote the conversion of pre-miR-1246 to mature miR-1246 mRNA, and miR-1246 targets CCNG2 and inhibits its expression	Bi et al. (2021b)
	Up	Promote cell proliferation and migration, and inhibit cell apoptosis	As an independent factor affecting ovarian cancer prognosis and correlated with ovarian cancer-associated oncogene m6A enrichment	Ma et al. (2020)
	Up	Promote migration and invasion	Stabilize TRPC3 mRNA expression *via* m6A modification	Shen et al. (2022b)
	Up	Promote cell proliferation, tumor growth, and invasion	Stimulate AXL translation and EMT	Hua et al. (2018)
	Up	Inhibit cell apoptosis and promote the cell cycle	Increase the level of KRT8, and decrease the level of FAS	Yang et al. (2022)
	Up	Inhibit cell apoptosis and promote invasion	Increase activation of the AKT signaling pathway and the expression of the downstream effector Cyclin D1	Liang et al. (2020b)
	Up	Promote cell growth and development, metastasis and invasion, and weaken cisplatin sensitivity	Mettl3/YTHDF2 axis represses tumor suppressor gene IFFO1 expression and promotes mRNA degradation	Zhang et al. (2023)

Compared to normal epithelial cells, Mettl3 is highly expressed in OC cells and is significantly associated with a variety of adverse clinicopathological features. Mechanistically, the Mettl3-mediated increase in the receptor tyrosine kinase AXL translation promotes epithelial-mesenchymal transition (EMT), leading to cellular proliferation, migration, invasion, and tumor formation (Hua et al., 2018). Besides, it has been experimentally demonstrated that Mettl3 activates the AKT pathway in OC cells. The investigators found that low expression of Mettl3 may facilitate OC cell apoptosis through the mitochondrial apoptotic pathway and inhibit cancer cell invasion by reducing activation of the AKT signaling pathway and the expression of the downstream effector Cyclin D1 (Liang et al., 2020b). Like its role in CC, silencing Mettl3 in OC increases apoptosis and arrests the cell cycle in the G0/G1 phase (Yang et al., 2022).

In summary, all current studies suggest that high expression of Mettl3 in OC causes adverse biological behaviors and promotes tumor development. Regrettably, despite the promising role of Mettl3 as a therapeutic target in OC, no treatments targeting Mettl3 have been developed to date. Further exploration of the underlying mechanisms of Mettl3 in OC is warranted, and there is a need for the active design and development of Mettl3 inhibitors to provide potential benefits for patients.

## The role of Mettl3 in endometrial cancer

EC, ranked among the three most prevalent gynecologic malignancies, was responsible for 41,700 newly diagnosed cases globally in 2020. Furthermore, its incidence has demonstrated a gradual increase over a 30-year period, with an overall rise of 132% (Gu et al., 2021; Sung et al., 2021). The current treatment plan for EC is mainly surgery combined with adjuvant therapies, such as radiotherapy, chemotherapy, targeted therapy, and hormone therapy (Aoki et al., 2020). ECs are often detected at an early stage, enabling a diagnosis and successful treatment. With a 5-year survival rate surpassing 95%, the prognosis for most early EC patients is optimistic (McMeekin, 2009). However, as the stage progresses, the 5-year survival rate decreases dramatically. Thus, there is an urgent need to investigate the molecular mechanisms of EC and optimize patient prognosis (Colombo et al., 2016). Previous studies have demonstrated that m6A regulatory proteins are implicated in multiple biological behaviors closely related to disease progression of EC, including proliferation, migration, and invasion, and they serve as key players in the activation of multiple signaling pathways (Li et al., 2021b; Zhang et al., 2021b; Ralser et al., 2022).

While there have been some studies investigating the role of Mettl3 in EC, the number of studies is relatively limited compared to other gynecological malignancies. This may be due to the status that endometrial cancer is less common than CC and OC, and the understanding of the molecular mechanisms underlying endometrial cancer is still incomplete. However, the studies conducted so far suggest that Mettl3 may play a key role in EC tumorigenesis and progression, and therefore further research is warranted. Liu et al. used immunohistochemical staining to compare tumor samples from patients with EC with adjacent tissues, and they found that Mettl3 expression was downregulated in tumor samples. Inhibited expression of Mettl3 was detected along with the loss-of-function mutation of Mettl14 and reduced m6A levels in EC tissues, and it was correlated with the proliferative effects of tumor cells (Liu et al., 2018b). Mechanistically, reduced m6A methylation affects multiple AKT pathway components by downregulating the AKT pathway negative regulator PHLPP2, upregulating the positive regulator mTORC2, and finally activating the AKT pathway. This ultimately promotes the tumorigenicity of EC cells and leads to disease progression (Table 3) (Liu et al., 2018b). Similarly, Ruan et al. demonstrated that Mettl3 is very closely related to activation of the AKT pathway. The authors significantly reduced m6A methylation by silencing Mettl3 expression in EC cell lines while recruiting YTHDF1 and YTHDF2 to regulate PAPPA and IGFBP4 at the translational level. Upregulated PAPPA further reduced the expression level of IGFBP4 by hydrolyzing IGFBP4, thereby decreasing the inhibition of IGF1, activating the AKT pathway, and eventually promoting the growth and development of EC cells (Ruan et al., 2022). Specifically, in EC, Mettl3 has been found to directly bind to the small nucleolar RNA SLERT and increase the m6A level of brain-derived neurotrophic factor (BDNF) mRNA. The m6A site on BDNF mRNA is then recognized and bound by the RNA-binding protein IGF2BP1, which enhances the stability of BDNF mRNA and subsequently activates BDNF/TRKB signaling. This activation induces epithelial-mesenchymal transition (EMT) and ultimately leads to lung metastasis of EC cells (Tian et al., 2023).

**TABLE 3:** The role of Mettl3 in endometrial cancer.

Cancer species	Expression	Biological function	Mechanism	Reference
endometrial cancer	Down	Promote cell proliferation and tumorigenicity	Decrease expression of the negative AKT regulator PHLPP2 and increase expression of the positive AKT regulator mTORC2	Liu et al. (2018b)
	Down	Promote cell proliferation and tumor formation	Reduce the expression level of IGFBP4, decrease the inhibition of IGF1, activae the AKT pathway	Ruan et al. (2022)
	Up	Promote cell migration and invasion, and induce EMT	Enhances the stability of BDNF mRNA and activates BDNF/TRKB signaling	Tian et al. (2023)

Although evidence suggests that dysregulated m6A methylation is involved in the development and progression of EC, the exact association between m6A methylation and EC remains unclear (Zhang et al., 2021a). Interestingly, the level of m6A methylation, both elevated and reduced, played a cancer-promoting role in EC cells (Ruan et al., 2022; Tian et al., 2023). This phenomenon may result from differences in the genomic sequence profiles of different cell lines or perhaps from the diversity of m6A methylation’s functions. Exploring the relationship between the two factors may illuminate novel insights into the diagnosis and treatment of EC, ultimately benefitting a massive number of patients and enhancing the prognosis of the disease.

## The clinical significance of METTL3 in gynecologic cancers

Mettl3 affects various biological behaviors of tumor cells by regulating m6A methylation, such as cell proliferation, apoptosis, invasion, and metastasis (Zeng et al., 2020). Additionally, Mettl3 can regulate tumor cell sensitivity to chemotherapy and immunotherapy, making it a potential target for cancer therapy (Xu and Ge, 2022). At the same time, the expression level of Mettl3 is closely related to the clinical outcome and therapeutic efficacy. Mettl3 is an independent factor affecting the survival of CC patients, especially DFS as well as OS, and it is closely associated with the disease stage (Ni et al., 2020; Wang et al., 2020d). Compared to patients with early-stage CC, Mettl3 expression levels were significantly higher in patients with advanced CC, suggesting a gradual increase in Mettl3 during the process of tumor malignancy (Hu et al., 2020; Shen et al., 2022a). The occurrence of CC is closely associated with HPV infection status, and previous studies have investigated the correlation between Mettl3 and HPV infection status, potentially offering a new avenue for the prevention, early diagnosis, and treatment of CC (Goodman, 2015; Yu et al., 2022). In addition, developing immune checkpoint inhibitors in recent years has significantly benefited cancer patients. A study by Yu et al. found that Mettl3 was correlated with the expression level of immune checkpoints; and it revealed that in mice, Mettl3 inhibitors combined with anti-PD-1 therapy could slow down tumor progression (Yu et al., 2022). This has given us a novel insight into the clinical treatment of patients with CC. Further, Mettl3 is also related to the chemosensitivity of cisplatin, and it might be possible to determine the chemotherapeutic dose of cisplatin by detecting the expression of Mettl3 in patients’ tumor tissues (Li et al., 2021a). In OC, the expression level of Mettl3 as an independent factor was similarly linked to patient prognosis and immunotherapy response (Tan et al., 2022). Mettl3, which is highly expressed in OC, is associated with the tumor grade, size of the tumor, lymph node metastasis, distant metastasis, FIGO stage, and overall survival rate (Hua et al., 2018; Liang et al., 2020b). Unfortunately, to date, no studies have pointed any significant association between Mettl3 expression and clinical features of EC. Therefore, further research is needed to explore the potential relationship between Mettl3 and the development and progression of EC.

Due to the broad involvement of m6A methylation in numerous stages of tumorigenesis, the regulatory proteins of m6A are increasingly being recognized as promising therapeutic targets (Boriack-Sjodin et al., 2018). According to the above-described summary, Mettl3 mainly plays an oncogenic role in gynecological malignancies, although there are a few reports of its tumor-inhibiting effects. The development of drugs targeting Mettl3 is still in the early stages, and at present, only Mettl3 inhibitors are available for experimental use. The recently revealed mechanism of Mettl3 inhibitor is a competitive binding of SAM, which can be categorized into nucleotide and non-nucleotide species. Nucleoside inhibitors were the first to be developed. Researchers used the AutoDuck program to screen 70 compounds from more than 4,000 compounds that could bind tightly to the SAM binding site through hydrogen bonding interactions on the NH group of the backbone (Morris et al., 2009). Subsequently, seven compounds were obtained from these 70 compounds by the HTRF method and detection of reaction products, and an adenosine derivative compound was found to have effective inhibitory activity against Mettl3 as well as potent pairing efficiency (Wiedmer et al., 2019; Bedi et al., 2020). However, due to the disadvantages of nucleoside inhibitors, such as weak cell permeability and poor selectivity for other methyltransferases, attention has shifted towards non-nucleoside inhibitors (Stein et al., 2018). Innovative material disciplines are developing rapidly, and polymeric nanomaterials are being used in a range of applications, including medicine. The polymer nanometer inhibitor UZH1a binds to the adenosine portion of SAM by van der Waals forces and hydrogen bonding forces formed with the polar group on Mettl3 (Zhao et al., 2020b). Compared to nucleoside inhibitors, UZH1a not only has high selectivity and better targeting performance, but it also has excellent physicochemical properties. In addition, Caflisch et al. optimized UZH1a and developed a small molecule inhibitor with superior properties, UZH2 (Dolbois et al., 2021). Further, STM2475, the first-in-class non-nucleoside Mettl3 inhibitor, has demonstrated its efficacy in the treatment of malignant tumors *in vitro*, which is an exciting achievement in the field of Mettl3 research (Yankova et al., 2021). However, the current research on Mettl3 inhibitors is mostly focused on hematological malignancies, and there are no research reports of Mettl3 inhibitors in gynecologic tumors; thus, further investigation is warranted. While, with ongoing research, it is possible that other types of drugs targeting Mettl3 could be developed in the future.

## Conclusion and perspectives

Cancer is a daunting global health challenge, and its unique biological behaviors, such as infinite proliferation, immune escape, angiogenesis, and individual tumor microenvironment, have resulted in numerous difficulties in its diagnosis and treatment (Hanahan and Weinberg, 2011). M6A methylation, the most prevalent type of mRNA modification in eukaryotic, is a ubiquitous modification of mRNA involved in nearly every aspect of mRNA metabolism, including translation, maturation, degradation, and folding (Liu and Gregory, 2019). It has been well established that m6A methylation is involved in many biological behaviors associated with cancer, and it plays a vital role in these biological behaviors, including promoting tumor cell proliferation, cell viability, invasion, and metastasis; regulating differentiation; and inhibiting apoptosis (He et al., 2019; Ma et al., 2019). The process of m6A methylation is regulated by three types of regulators: “writers,” “erasers,” and “readers,” which interact with one another to achieve precise control of m6A modification. The Mettl3 catalytic subunit is the main component of the writer, and it plays a unique catalytic role (Chen et al., 2019). This article reviewed new developments in the study of Mettl3 in gynecologic malignancies in recent years, with a focus on cell proliferation, invasion, migration, and activation of cancer-related pathways (Zhang and Liu, 2022). In addition to directly regulating cancer cell biological behavior, Mettl3 has been found to be correlated with HPV infection status, an established causative factor of CC (Yu et al., 2022). Further, the Warburg effect, a characteristic of malignant cells, has likewise been shown to be profoundly influenced by Mettl3 (Wang et al., 2020d; Du et al., 2022). Mettl3 is also involved in several specific biological behaviors of OC and EC. Given the extensive and diverse roles played by Mettl3 in gynecologic malignancies, further investigation into its potential mechanisms in tumor progression is warranted to identify novel therapeutic targets that could offer hope to cancer patients globally.

Although this manuscript focuses on the role of Mettl3 in gynecologic malignancies, m6A methylation is a dynamically regulated process, and other regulatory proteins’ functions are equally important (Lan et al., 2019). Notably, the coexistence of Mettl3 in multiple types of cancer with diametrically opposed effects on tumor progression is a question that deserves thoughtful consideration. For example, in CC, Fei Ji et al. found that Mettl3 could promote the development of CC. At the same time, the results presented by Ruyi Li et al. suggested that Mettl3 could inhibit the action of oncogenes (Li et al., 2021a; Ji et al., 2021). These findings suggest the importance of investigating specific regulatory proteins and their interactions when studying m6A methylation. The heterogeneity of the malignancy may be responsible for this phenomenon and is often the culprit of clinical treatment failure. This observation indicates the need to transition towards personalized and precise approaches in both basic research and clinical management in the future. Although sporadic Mettl3 inhibitor studies have been published, there is still a gap in its application in gynecologic malignancies. More in-depth studies are required to investigate the specific role played by Mettl3 in malignancies, and it may shed light on new therapeutic approaches for gynecologic tumors.

In summary, the m6A methylation process, regulated by a writer-eraser-reader complex, plays a significant role in gynecologic malignancies. Among the regulatory proteins, Mettl3, a writer, has been found to have multiple roles and extensively affects various biological behaviors of tumor cells. Its dysregulation has been associated with oncogenic transformation, metastasis, chemoresistance, and poor prognosis in gynecologic malignancies. However, current research on Mettl3’s role in tumors may only scratch the surface, and the development of drugs targeting Mettl3 is still in its infancy; there is a need to study its mechanism of action in depth and work on designing new treatments to provide more benefits to a great quantity of patients with gynecologic malignancies.
